# Temperature effects on the internal conversion of excited adenine and adenosine†‡

**DOI:** 10.1039/d3cp03234e

**Published:** 2023-09-22

**Authors:** Ritam Mansour, Josene M. Toldo, Saikat Mukherjee, Max Pinheiro, Mario Barbatti

**Affiliations:** a Aix Marseille University, CNRS, ICR Marseille France josene-maria.toldo@univ-amu.fr mario.barbatti@univ.amu.fr https://www.barbatti.org; b Institut Universitaire de France 75231 Paris France

## Abstract

This work aims to elucidate the dependence of the excited-state lifetime of adenine and adenosine on temperature. So far, it has been experimentally shown that while adenine's lifetime is unaffected by temperature, adenosine's lifetime strongly depends on it. However, the non-Arrhenius temperature dependence has posed a challenge in explaining this phenomenon. We used surface hopping to simulate the dynamics of adenine and adenosine in the gas phase at 0 and 400 K. The temperature effects were observed under the initial conditions *via* Wigner sampling with thermal corrections. Our results confirm that adenine's excited-state lifetime does not depend on temperature, while adenosine's lifetime does. Adenosine's dependency is due to intramolecular vibrational energy transfer from adenine to the ribose group. At 0 K, this transfer reduced the mean kinetic energy of adenine's moiety so much that internal conversion is inhibited, and the lifetime elongated by a factor of 2.3 compared to that at 400 K. The modeling also definitively ruled out the influence of viscosity, which was proposed as an alternative explanation previously.

## Abstract 2

Temperature has a strikingly different impact on adenine and adenosine photodynamics. Surface hopping reveals the reason underlying it.

## Graphical abstract

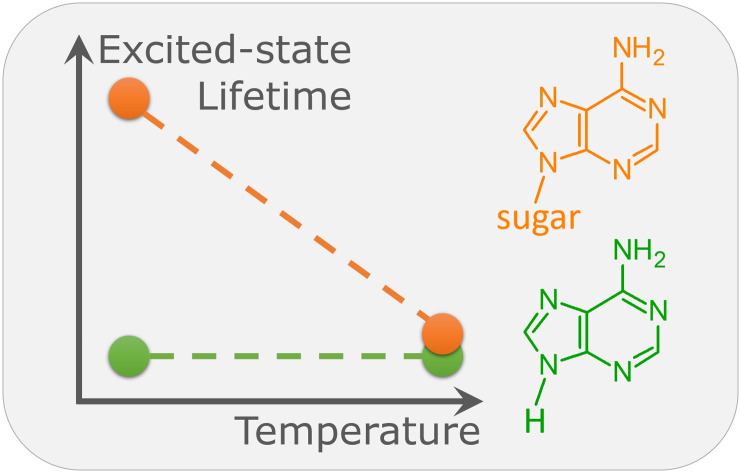

## 1. Introduction

It is well-established experimentally and computationally that all canonical nucleobases and nucleosides undergo internal conversion to the ground state on a picosecond scale after UV photoexcitation, either in the gas phase or in solution.^1–3^ This ultrafast internal conversion is dominated by ring puckering deformations bringing the molecule to conical intersection regions. However, one aspect that has received little attention is the dependence of the internal conversion mechanisms on environment's temperature.

The effect of the temperature on the ultrafast dynamics of nucleobases and nucleosides was experimentally investigated by West *et al.*^4^ using time-resolved spectroscopy. They compared the dynamics of thymine, thymidine, and thymine dinucleotide in a mixture of methanol and water over a range of 100 to 300 K. The ππ* excited-state lifetime of thymine is independent of temperature, remaining at around 0.6 ps. In the case of thymidine and thymine dinucleotide, the excited-state lifetimes revealed an Arrhenius-type dependence on the temperature [ln(*τ*) ∝ *T*^−1^], dropping from 2.0 to 0.8 ps in the nucleoside when the system was heated from 100 to 300 K. In the dinucleotide, the drop was from 3.7 to 1.2 ps. West *et al.*^4^ also compared the dynamics of 9-methyl-adenine and adenosine under the same conditions. Once again, the nucleobase ππ* excited-state lifetime is found to be independent of temperature, remaining at about 0.4 ps. In contrast, adenosine's excited-state lifetime dropped from 1.3 ps at 100 K to 0.5 ps at 300 K. Nevertheless, the dependence on the temperature does not follow an Arrhenius behavior. After dropping between 100 and 166 K, the excited-state lifetime remains constant at 0.5 ps between 166 and 300 K.

For thymine derivatives, West *et al.*^4^ attributed the temperature-dependent excited-state lifetime to the intramolecular vibrational energy transfer from the nucleobase to the deoxyribose ring and discarded any role of solvent viscosity. Vibrational energy dissipation competes with internal conversion, increasing the time needed for the excited-state population to access the conical intersection with the ground state. On the other hand, West *et al.* claimed that viscosity effects may be more pronounced for adenine derivatives. The long excited-state lifetime of adenosine at 100 K would result from medium viscosity, inhibiting out-of-plane modes needed for internal conversion. This viscosity argument, as those authors recognized, is not problem-free. First, because the solvent freezes near 150 K, the viscosity increase when adenosine is cooled from 166 to 100 K, which would have a substantial impact to cause a mere 2.5-factor increase in the lifetime. Second, it is unclear why viscosity would only inhibit out-of-plane distortions in adenosine but not in adenine, given that they share the same types of conical intersections.^5^

**Fig. 1:**
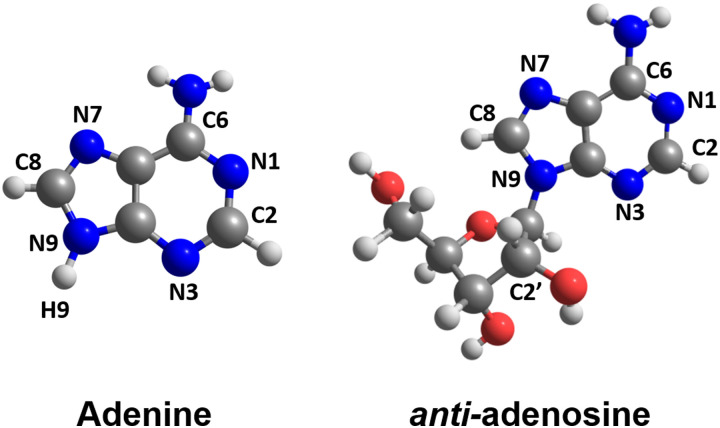
Molecular structures of 9H-adenine and *anti*-adenosine investigated in this work.

Our work aims to clarify this situation, explaining the reason underlying adenine and adenosine's excited-state lifetime dependencies on temperature. To access this information, we computed four sets of surface hopping simulations for these molecules (Fig. 1) at different temperatures. Our goal is not precisely to reproduce the experimental setup but to include the crucial elements to describe the photodynamics, as the excited-state lifetime, the vibrational energy distribution, and the relevant nuclear vibrational modes.

Thus, we adopted the following simplifications compared to the experiments:

First, our dynamics simulations are set in the gas phase and not in a liquid solvent. This may seem a radical approximation, but the coupling between the nucleobase and the ribose is much stronger than between the nucleoside and the solvent. As West *et al.*^4^ observed, this difference in coupling strength leads to a much faster intramolecular vibrational energy transfer than intermolecular vibrational energy transfer, and the former tends to dominate the effects on the excited-state lifetime. The exception is when solvent viscosity inhibits the internal conversion mechanism. If that was the case, our simulations would fail to detect any temperature dependence on the excited-state lifetime of adenosine.

Second, we assumed a collisionless regime, meaning that the gas concentration is so low that there are no molecular collisions while the molecule is excited. This approximation implies that the initial conditions determine the available thermal energy, but the excited-state dynamics runs with constant total energy. Once more, this hypothesis is only sensible if intramolecular coupling dominates over intermolecular coupling.

Third, owing to the high dynamics computational costs, we only simulated adenine and adenosine at two temperatures each. Therefore, we can detect temperature effects but cannot determine their functional dependencies, although we can indirectly infer them. Furthermore, our simulations were run at 0 and at 400 K, a wider temperature range than in the experiments, to enhance any thermal effects.

Finally, the electronic structure method adopted to feed the surface hopping simulations allows the detection of internal conversion to the ground state but it does not allows the propagation of the hot ground-state dynamics. Thus, our analysis is intrinsically limited to the excited-state processes, and we will not discuss the ground-state relaxation.

Even with all these approximations, our results align with the experiments, showing a temperature-independent excited-state lifetime for adenine and a much longer excited-state lifetime at a low temperature than at a high temperature for adenosine. These temperature-dependence patterns were entirely shaped by intramolecular vibrational energy transfers, with no reliance on viscosity or other solvent effects.

## 2. Methodology

### 2.1 Including temperature effects on the dynamics

In the collisionless regime assumed in this work, the thermal energy is determined before photoexcitation and does not change during the excited-state dynamics. Thus, the system is effectively propagated as a microcanonical ensemble with constant total energy. This assumption also implies that all information about temperature is encompassed in the initial conditions. This subsection details the procedures we adopt to create them.

Surface hopping simulation requires selecting initial geometries, momenta, and initial states. A common way to obtain these values is using a probability distribution function, where they are generated randomly based on a quantum harmonic oscillator distribution.^6,7^ Assuming that the molecule is initially at the minimum of the ground state, and the potential energy surface around it is harmonic, geometries and momenta follow a Wigner probability distribution for the thermal population of the vibrational states^6,8^1
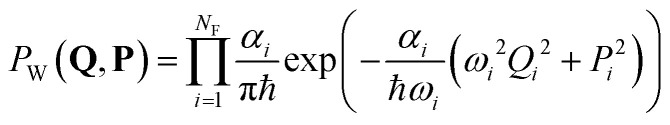
where *N*_F_ = 3*N*_at_ − 6 is the number of degrees of freedom of a molecule with *N*_at_ atoms, *Q*_*i*_ = *μ*^1/2^_*i*_*q*_*i*_ and *P*_*i*_ = *μ*^−1/2^_*i*_*p*_*i*_ are the mass-scaled coordinate and momentum for each normal mode *i* with coordinate *q*_*i*_, momentum *p*_*i*_, reduced mass *μ*_*i*_, and angular frequency *ω*_*i*_. In our simulations, the effect of the temperature (*T*) is introduced *via* the variable,2
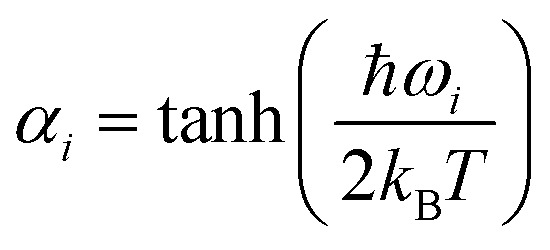
which controls the distribution width for each normal mode with angular harmonic frequency *ω*_*i*_. When *T* = 0, *α*_*i*_ = 1, the energy is equal to the zero-point energy, and the distribution is sharp; when *T* gets higher, 〈*E*〉 has a smaller value, and the Gaussian distribution becomes broader. This sampling prescription creates an ensemble of initial conditions with mean total energy3
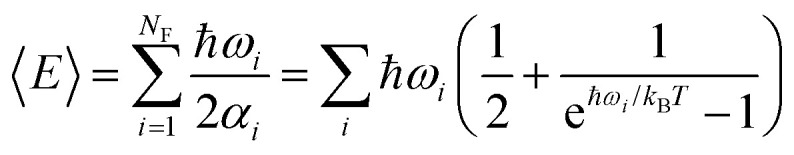
However, because of the independent random sampling of **Q** and **P** in this uncorrelated distribution, the total energy of the ensemble of molecules has a large dispersion of values around the mean,^9^ with the standard deviation given by4
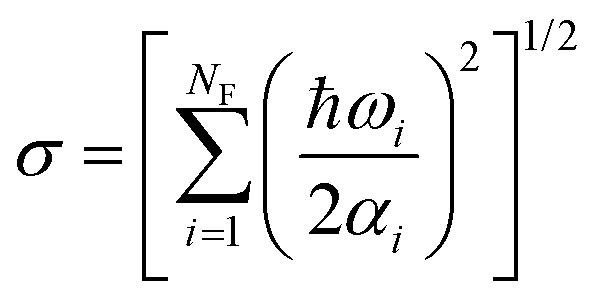
Alternatively, it is possible to adopt a correlated distribution. In this case, we randomly create a nuclear geometry and choose the corresponding momentum to match a unique total energy value. By construction, such a distribution has zero total energy dispersion.^10^ Thus, to get a sharp distribution of the energies around 〈*E*〉, we use a correlated sampling by integrating the Wigner distribution over the momenta5
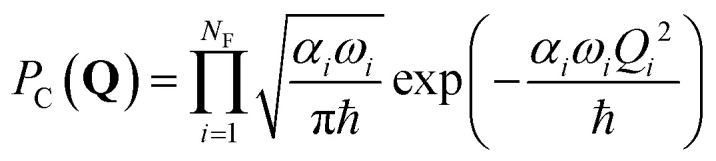
For each random geometry, the momentum of each normal mode is such that the total energy stored should be its own temperature-corrected zero-point energy. Thus,6
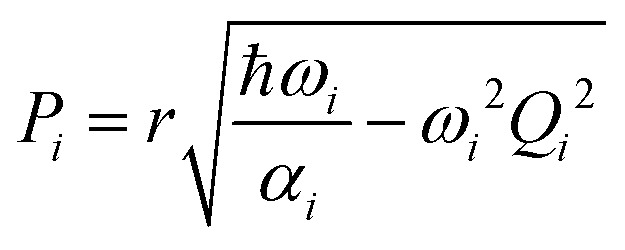
where *r* is either +1 or −1, chosen randomly. After sampling, the normal-mode coordinates and momenta are converted to Cartesian coordinates and momenta. To account for the anharmonicity introduced by the quantum-chemical calculations, the Cartesian velocities are rescaled as:7
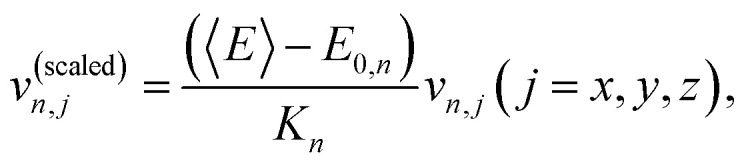
rendering a sharp distribution around 〈*E*〉, where *K*_*n*_ is the total harmonic kinetic energy of the sampled geometry *n* and *E*_0*,n*_ is the ground-state potential energy for this geometry computed using the quantum-chemical method.

When studying the temperature effect on dynamics, we could use either the uncorrelated or correlated approach. However, if we used the uncorrelated sampling, we would not be sure whether any difference between the dynamics results at different temperatures was due to the temperature or total energy spreading. Thus, we decided to proceed using correlated sampling.

Zobel *et al.*^11^ proposed an alternative way to sample temperature-dependent Wigner distributions and applied it to investigate the temperature effects in the excited-state dynamics of 2-nitronaphthalene. Instead of sampling initial coordinates and momenta using eqn (1), their approach first samples Boltzmann-distributed vibrational states and then coordinates and momenta for each of these states.

### 2.2 Computational details

All simulations were done for 9H-adenine and the *anti*-conformer of adenosine as indicated in Fig. 1. We exclusively report the dynamics of this *anti*-isomer to avoid the effect of the intramolecular hydrogen bond between the ribose and adenine moieties that appear in the *syn*-isomer.^5,12^ The surface hopping dynamics of *syn*- and *anti*-isomers of adenosine was discussed in a recent investigation by our group.^5^

The ground-state equilibrium geometries were optimized using the Møller–Plesset to the second-order (MP2) level of theory and the SV(P) basis set.^13^ Excitation energies were calculated using the resolution-of-the-identity algebraic diagrammatic construction to the second-order method [RI-ADC(2)]^14–18^ with the same basis set. This method has successfully described adenine derivatives in both statical and dynamical regimes.^5,19–22^ All electronic structure calculations were performed using Turbomole v. 7.3.^23^ For optimizing S_1_/S_0_ crossing points, an in-house modified version of the CIOpt program^24^ was used. Linearly interpolated pathways using internal coordinates were computed between the S_1_ minimum and the S_1_/S_0_ intersections.

The initial conditions for the dynamics were generated using the correlated quantum harmonic oscillator distribution as implemented in Newton-X CS (v. 2.5 build 05; https://www.newtonx.org).^25^ A set of 500 random molecular geometries was created. The velocities were generated according to the related procedure described in Section 2 for a sharp distribution around the mean total energy value.

The absorption spectra of adenine and adenosine at 0 and 400 K were simulated using the nuclear ensemble approach.^26^ Vertical excitation energies and oscillator strengths for the first four electronic transitions were computed using RI-ADC(2)/SV(P) for each geometry in the ensemble. For each temperature, the initial conditions for the dynamics were chosen according to the simulated spectrum and filtered by their oscillator strength within an energy window of 5.1 ± 0.1 eV. The number of trajectories starting in each state is given in Table 1.

**Table 1:** Characterization of the four sets of dynamics. Temperature and the number of trajectories starting in the individual states (S_1_ to S_3_). The excitation energy window was 5.1 ± 0.1 eV, and the total number of trajectories was 50 for each set

Set	Molecule	*T* (K)	Number of trajectories starting in		
			S_1_	S_2_	S_3_
1	Adenine	0	10	37	3
2	Adenosine	0	14	32	4
3	Adenine	400	10	31	9
4	Adenosine	400	5	22	23

Photoinduced nonadiabatic dynamics were simulated using the decoherence-corrected^27^ fewest-switches surface hopping (DC-FSSH) approach^28^ as implemented in Newton-X CS. The trajectories were propagated using the local diabatization algorithm^29,30^ with the orbital derivative approach to get the wave function overlap matrices.^31^ The classical equations of motion were integrated using the Velocity Verlet^32^ algorithm at each 0.5 fs time step, and the total energy was kept constant during the simulations. The quantum chemical calculations were performed on the fly at the RI-ADC(2)/SV(P) level using Turbomole. The simplified decay approach for mixing^27^ was applied for decoherence corrections using the standard 0.1 a.u. parameter. The velocity was adjusted along the momentum direction using a size-extensivity correction to prevent an artificial excess of back hoppings.^33^ The momentum direction was kept constant in the case of frustrated hoppings.

For each set, 50 trajectories were propagated up to 2 ps. Only hoppings between excited states were computed. Due to the limitation of RI-ADC(2) to deal with multireference ground states, trajectories were stopped whenever their S_1_/S_0_ energy gap dropped below 0.15 eV. The time this threshold was reached was taken as an indicator of internal conversion time to the ground state, a common practice in surface hopping dynamics using single-reference methods.^19,34–36^

Statistical analysis was performed using ULaMDyn (https://www.ulamdyn.com). Normal modes analysis over the trajectories was done using the corresponding module available in Newton-X CS.

## 3. Results

### 3.1 The excited states of adenine and adenosine

We begin our discussion by characterizing the first excited states of adenine and adenosine. Both molecules have similar S_1_, S_2_, and S_3_ excitation energies and oscillator strengths (Table 2). S_1_ is a dark nπ* state at the ground state geometry, while S_2_ is the first allowed ππ* transition (ESI,‡ Section S1). It has been shown that ADC(2) satisfactorily predicts excitation energies and reaction pathways for adenine and adenosine when compared to a higher level of theory (see ESI,‡ Section S1 for comparison with different levels of theory).

**Table 2:** Excitation energies and oscillator strengths (*f*) for adenine and adenosine at the S_0_ minimum at the ADC(2)/SV(P) level

State	Adenine		Adenosine	
	Energy (eV)	*f*	Energy (eV)	*f*
S_1_	5.16	0.001	5.13	0.000
S_2_	5.34	0.060	5.25	0.091
S_3_	5.52	0.269	5.45	0.283

At 0 K, the simulated absorption spectrum peaks at 5.39 eV for adenine and 5.31 eV for adenosine (ESI,‡ Section S2). Apart from a small redshift, no significant changes are observed in the spectrum simulated at 400 K; its maximum is 5.37 eV for adenine and 5.25 eV for adenosine. We opted for selecting the initial conditions for the dynamics within the same spectral window (5.1 ± 0.1 eV) in all cases, slightly below the spectral band maximum. This choice was made based on the experimental setup by West *et al.*,^4,37^ wherein adenine and adenosine were photoexcited at 4.68 eV (265 nm) for all the temperatures, also slightly below adenine's and adenosine's absorption maximum at pH ∼ 7.0 and 4.77 eV.^38,39^

**Fig. 2:**
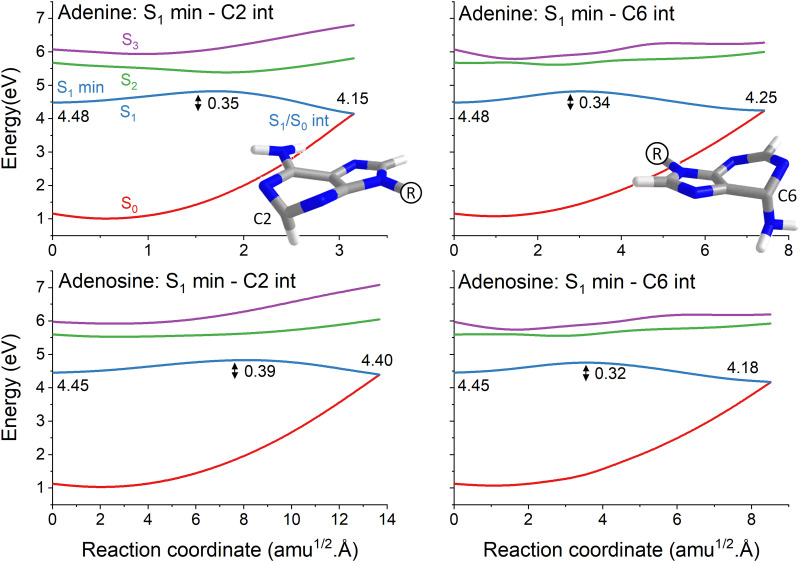
Reaction pathways between the S_1_ minimum and the two main adenine (top) and adenosine (bottom) S_1_/S_0_ intersections. These intersections are characterized by ring puckering at C2 (left) or C6 (right). Four states are shown from S_0_ to S_3_. The reaction path is a linear interpolation between the S_1_ minimum and the S_1_/S_0_ state intersection. Their respective energies (in eV) are given in each graph. The energy barrier from the S_1_ minimum is indicated too. Mind the different abscissa scales in each graph.

The topography of the main reaction pathways for S_1_/S_0_ internal conversion is illustrated in Fig. 2. It shows the potential energy profiles connecting the S_1_ minimum to the C2-puckered and C6-puckered S_1_/S_0_ intersections, the most common deactivation channels in adenine and adenosine.^5,40^ All cases have an energy barrier on S_1_ of about 0.3 to 0.4 eV. These values, however, should be overestimated using the linear interpolation procedure. The nuclear distortion to reach the C6-puckered intersection is similar in both molecules (about 7 to 8 amu^1/2^ Å). Nonetheless, the distortions to reach the C2-puckered intersection are largely uneven in adenine (3 amu^1/2^ Å) and adenosine (14 amu^1/2^Å). Such larger distortion in adenosine has been confirmed in ref. 5 by checking the S_1_/S_0_ intersection geometries reached during dynamics and is also verified in our results discussed in Section 4.3.

### 3.2 Excited-state lifetimes

**Fig. 3:**
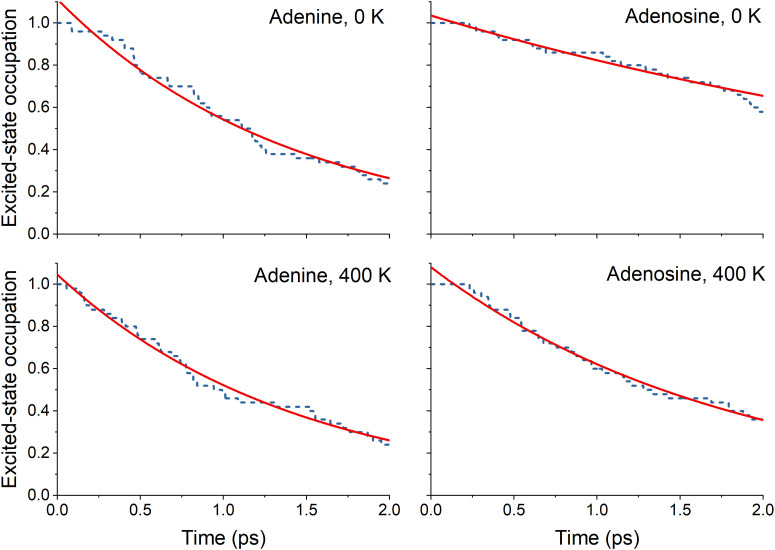
Excited-state occupation (fraction of trajectories in the exited states) as a function of time for adenine and adenosine at 0 and 400 K. The red line represents the fitted function used (eqn (8)). The fitted parameters are given in Table 3.

The nonadiabatic dynamics simulations starting from the bright state were run up to 2 ps for both molecules at 0 and 400 K. For all four sets of simulations, the S_3_ and S_2_ population is transferred to the S_1_ in the first femtoseconds of the simulation, as revealed by the state's occupation as a function of time (ESI,‡ Section S3). This nonadiabatic transfer corresponds to a relaxation of the ππ* state. The excited-state lifetime is obtained from the total excited-state occupation (S_1_ + S_2_ + S_3_) shown in Fig. 3.

The excited-state lifetimes were obtained by fitting the fraction of the excited-state population decay using the exponential decay function

8 *f*(*t*) = *f*_∞_ + (1 − *f*_∞_)e^−(*t*−*τ*_1_)/*τ*_2_^,

where *τ*_1_ is the latency time to initiate the internal conversion, *τ*_2_ is the exponential decay time constant, and *f*_∞_ is the fraction of the population that does not decay with this time constant. The fitted parameters and the calculated lifetimes are presented in Table 3. The lifetime (*τ*) is given by *τ* = *τ*_1_ + *τ*_2_. At 0 K, the excited-state lifetime of adenine is 1.5 ps. For adenosine, the excited state lifetime is considerably longer (4.5 ps). At 400 K, both molecules exhibit similar lifetimes; 1.5 and 2.0 ps for adenine and adenosine, respectively.

**Table 3:** Lifetime (*τ*) predicted by surface hopping dynamics for adenine and adenosine computed at the ADC(2)/SV(P) level. *f*_∞_ converged to 0 in all cases. The error bars are for the 95% confidence interval

Molecule	*T* = 0 K			*T* = 400 K		
	*τ*_1_ (ps)	*τ*_2_ (ps)	Lifetime (ps)	*τ*_1_ (ps)	*τ*_2_ (ps)	Lifetime (ps)
Adenine	0.15	1.39	1.5 ± 0.4	0.07	1.44	1.5 ± 0.4
Adenosine	0.15	4.37	4.5 ± 1.2	0.14	1.81	2.0 ± 0.5

Considering the excited-state lifetimes shown in Table 3, we promptly see that increasing the temperature from 0 to 400 K does not affect adenine's excited-state lifetime but drastically impacts adenosine's excited-state lifetime. This finding qualitatively agrees with the experimental results obtained by West *et al.*^4^ In their work, the excited-state lifetime of adenine did not change when heating the molecule from 100 to 300 K. In contrast, this same heating reduced adenosine's lifetime by 2.5 times. In our simulations, this ratio is 2.3 between 0 and 400 K. This agreement is meaningful considering all the approximations adopted in our modeling, as discussed in the Introduction. Notably, the difference in absolute lifetime values (for instance, the theoretical prediction for adenine is 1.5 ± 0.4 ps, while the experiments^4^ give 0.5 ± 0.3 ps) can be attributed to the different environments. The experiments were performed in an ethanol/water (85 : 15) mixture, while the computational results were in the gas phase. In the gas phase, the experimental excited-state lifetime of 9-methyl-adenine (the species West *et al.* investigated) is 1.3 ± 0.1 ps after 267 nm excitation,^41^ which is comparable with our simulation.

### 3.3 Internal conversion mechanisms

It is well known that adenine has two main S_1_ to S_0_ internal conversion pathways, one corresponding to a ring puckering at atom C2 and another to a ring puckering at C6.^40^ These two pathways compete on the same time scale, and the precise splitting between them depends on the computational method.^42^ A third pathway enabled by N−H stretching (either in N9 or in the amino group) is also available,^43^ but it only plays a role at high excitation energies. In adenosine, in addition to the adenine's internal conversion pathways (excluding N9–H stretching, which is unavailable), an electron-driven proton transfer (EDPT) channel is also possible, which creates an S_1_/S_0_ intersection during hydrogen transfer between the ribose and adenine rings.^12^ As we stated above, we chose to work with an *anti*-conformer of adenosine to minimize the occurrence of EDPT and having adenine and adenosine undergoing internal conversion through similar pathways. Furthermore, ref. 36 shows that electron transfer from water to 7H-adenine opens a new S_1_/S_0_ internal conversion pathway but it requires an explicit water treatment. Nevertheless, this effect was restricted to the 7H isomer due to water molecules interacting with lone-pair electrons at N3 and N9. This internal conversion pathway does not occur in 9H-based derivatives, as those investigated in this work.

The partitioning of the deactivation pathways observed during the 2 ps of our dynamics is reported in Table 4. As expected, internal conversion occurs almost exclusively *via* puckered intersections with only small EDPT participation in adenosine. The N−H stretching mechanism was not observed. For adenine at 0 K, internal conversion may occur through C2 and C6 ring puckering mechanisms. C6 seems slightly favored, but the difference is not statistically significant, given the margins of error. Heating adenine from 0 to 400 K keeps the mechanisms partition remarkably unaltered. For adenosine at 0 K, both C2 and C6 puckering mechanisms are equally likely to occur. EDPT also contributes, but it is about six times less frequent than puckering. For adenosine at 400 K, C6 is favored over C2, but the difference may be statistically insignificant.

**Table 4:** Main deactivation pathways for S_1_/S_0_ internal conversion occurring within 2 ps and the remaining exited population at 2 ps predicted by surface hopping dynamics for adenine and adenosine. Margins of errors for a 95% confidence interval

Set	Mechanism breakdown at 2 ps (%)				
	C2 puckering	C6 puckering	Total puckering	EDPT	Excited
1. Adenine 0 K	34 ± 13	42 ± 14	76 ± 12	0	24 ± 12
2. Adenosine 0 K	20 ± 11	16 ± 10	36 ± 13	6 ± 7	58 ± 14
3. Adenine 400 K	30 ± 13	46 ± 14	76 ± 12	0	24 ± 12
4. Adenosine 400 K	24 ± 12	34 ± 13	58 ± 14	6 ± 7	36 ± 13

**Fig. 4:**
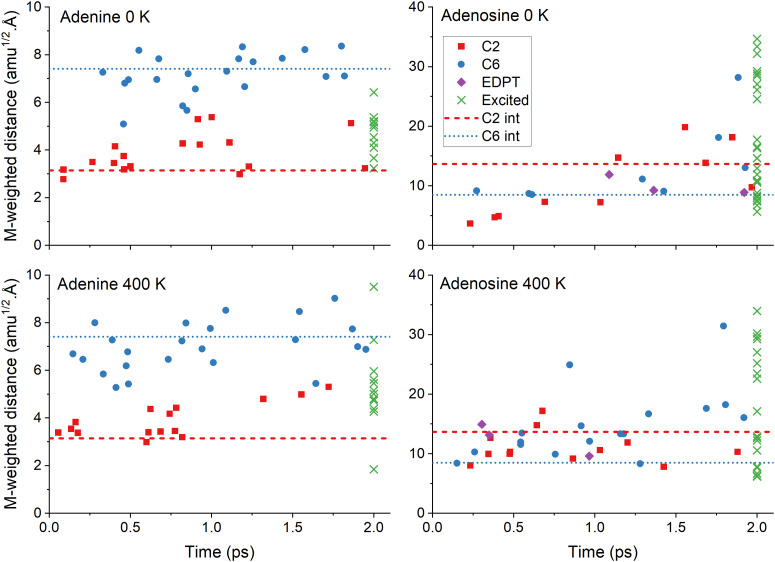
Mass-weighted distance of the S_1_/S_0_ internal conversion points as a function of time for the four simulation sets. The points are sorted by mechanisms: C2 puckering (red), C6 puckering (blue), and EDPT (violet). The latter is for adenosine only. The distance at 2 ps is also shown for the trajectories remaining in the excited states (green). The mass-weighted distance was computed between each final and the initial geometries of the corresponding trajectory. The horizontal lines show the distance for the minimum energy S_1_/S_0_ intersection (see Fig. 2). Mind the different scales in the vertical axes.

Fig. 4 delivers further details of the internal conversion mechanisms for each trajectory. In all cases, the internal conversion is distributed over the entire 2 ps range, as expected for an exponential population decay. Adenine and adenosine play in different mass-weighed scales because of the impact of the ribose group distortions on the latter. In adenine, the distribution of trajectories following either C2 or C6 puckering correlates with the mass-weighted distances. However, this is not the case for adenosine. The excited-state lifetime drop in adenosine from 4.5 to 2.0 ps when heated from 0 to 400 K (see Table 3) is due to an increase of C6-puckering trajectories in the 400 K simulation. Independent of the temperature, adenosine trajectories finished in the excited states span a wide range of distances up to 30 amu^1/2^ Å, reflecting the ribose group's significant rotational flexibility during the dynamics.

## 4. Discussion

### 4.1 Energy transfer at low and high temperatures

At this point, a central question arises: why does adenine's excited-state lifetime is independent of the temperature, while adenosine's excited-state lifetime shows a striking temperature dependence?

As discussed in detail in the Introduction, West *et al.*^4^ proposed that while the temperature effects on thymine derivatives should be caused by intramolecular vibrational energy transfer, they attributed it to viscosity effects in the case of adenine derivatives. Nevertheless, we can rule out viscosity because our simulations are done in the gas phase and still show temperature dependence. Therefore, vibrational energy transfer becomes the primary hypothesis to explain the phenomenon.

When adenosine is excited, the photon energy is deposited in the nucleobase, as seen in the plots for the electronic density difference between S_0_ and S_1_ (ESI,‡ Section S4). The excitation is exclusively localized in the adenine moiety in the pathway between the Franck–Condon region and the intersection region. Thus, when the excited-state relaxation process begins after the photoexcitation, the nucleobase acts as a reservoir for excess vibrational energy, which can flow to the ribose moiety, the colder part of the molecule.

**Fig. 5:**
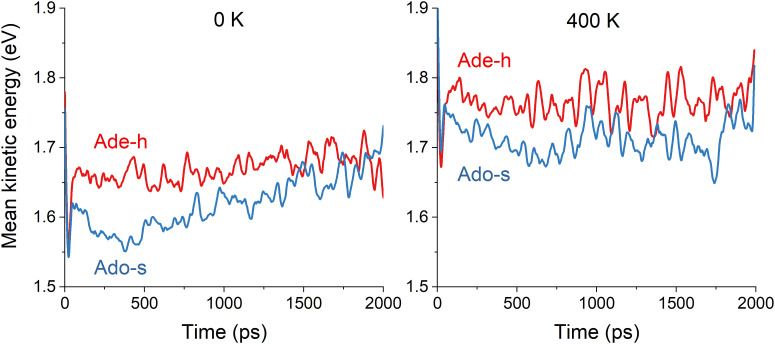
Mean kinetic energy of the adenine moiety without H9 (Ade-h) in adenine's simulations and without ribose (Ado-s) in adenosine's simulations. The mean values are computed over all trajectories in the excited states at each time step. Left: 0 K. Right: 400 K. The results are smoothed over 50 fs.

To investigate the intramolecular vibrational energy redistribution after the excitation still in the excited electronic state, we monitored the kinetic energy of the adenine moiety during the dynamics and averaged it over all trajectories. In adenine's simulations, we calculated the mean kinetic energy of the adenine moiety excluding H9 (we named it Ade-h for “adenine minus H”). In adenosine's simulations, we computed the mean kinetic energy of adenine excluding the ribose group (Ado-s for “adenosine minus sugar”). Thus, these quantities correspond to the same 14 atoms and can be directly compared. They are plotted in Fig. 5 for 0 and 400 K, smoothed over 50 fs to eliminate the strong oscillations associated with the hydrogen stretching vibrations.

At either 0 or 400 K, Ade-h maintains approximately constant kinetic energy (Fig. 5). At 0 K, this mean value is 1.67 eV; it is 0.1 eV higher at 400 K. On the other hand, Ade-s has significantly lower kinetic energy than Ade-h, it does not matter the temperature. At 0 K, the mean value is 1.62 eV, and at 400 K, it is 1.71 eV. The smaller kinetic energy of Ado-s than Ade-h implies that vibrational energy transfer to the ribose group occurs during the excited-state dynamics. After 1 ps, Ado-s’ kinetic energy at 0 K increases again, reaching similar levels as Ade-h.

These kinetic energy evolution patterns and the excited-state lifetimes draw a scenario where adenine at both temperatures and adenosine at 400 K have enough energy to reach the S_1_/S_0_ intersections without significant impediment. Adenosine at 0 K, however, loses too much energy to the ribose group, delaying its internal conversion to the ground state. Thus, these results imply that adenine moiety's kinetic energy in adenine at 0 K, adenine at 400 K, and adenosine at 400 K are always much superior to the energy barrier, allowing an access to the state intersection in roughly similar times between 1.5 and 2 ps. On the other hand, the adenine moiety in adenosine at 0 K does not seem to have enough energy to cross the barrier until it is resupplied later in the dynamics.

**Fig. 6:**
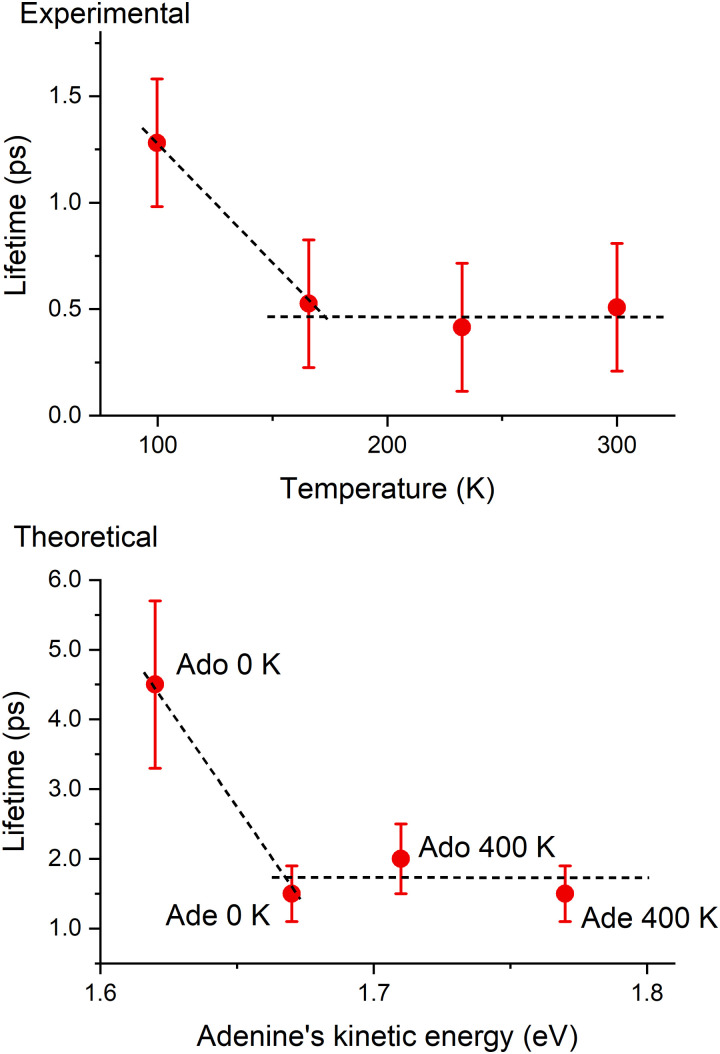
Top: Experimental excite-state lifetime of adenosine as a function of temperature reported in ref. 4. Bottom: current simulations of the excited-state lifetime of adenosine (Ado) and adenine (Ade) at different temperatures as a function of the mean kinetic energy of the adenine group. The dashed lines are only to guide the eyes.

The basis for West *et al.*'s^4^ claim that viscosity was the reason for the temperature dependence of adenosine's excited-state lifetime was the observation that although its lifetime was long at 100 K, it was short and constant between 166 and 300 K. This abrupt non-Arrhenius behavior, reproduced in Fig. 6 (top), was interpreted as a sign of a phase transition, which could be related to the solvent freezing at 150 K. Our analysis, however, points to a different cause for this effect. If adenine moiety's energy is greater than a certain level, the lifetime is insensitive to the temperature, leading to a similar profile, as shown in Fig. 6 (bottom).

### 4.2 Normal mode analysis

In the previous section, we discussed how the temperature effects on the excited state of adenosine were due to vibrational energy transfer between adenine and the ribose groups. Here, we want to determine which nuclear vibrational modes are responsible for these effects. This information can be gathered *via* normal mode analysis,^44,45^ which projects the trajectories on a normal mode basis and evaluates the contribution of each one at each time step. We highlight that in this analysis, the normal modes are only employed to project and describe the nuclear motion during molecular dynamics. They should not be mistaken by spectroscopic normal modes representing the vibrational motion around the minimum in the harmonic approximation. Thus, any normal mode basis can be used in normal mode analysis and applied even to potential energy surface regions where the harmonic approximations are not valid. The details of the procedure employed here are discussed in ESI,‡ Section S5.

The normal mode analysis was done for adenosine at 0 and 400 K using the ground state normal modes. We collected the standard deviation of each normal mode along each trajectory up to 2 ps simulation. This quantity is relevant because it informs how much the ensemble of trajectories deviates from the initial conditions in each mode. Thus, a normal mode with a prominent standard deviation is where the kinetic energy is deposited during dynamics.

Six normal modes are particularly relevant for the discussion that follows: Modes 1 and 2 with tiny wavenumbers of about 40 cm^−1^ corresponding to coupled torsional motions between adenine and ribose, Mode 4 at 102 cm^−1^ corresponding to the alkyl hydroxy twist, Mode 24 at 534 cm^−1^ with the shearing of the pyrimidine ring, Mode 33 at 755 cm^−1^ coupling adenine breathing to ribose shearing, and Mode 34 at 777 cm^−1^ with the coupled adenine-ribose breathing. These normal modes are illustrated in ESI,‡ Section S6.

**Fig. 7:**
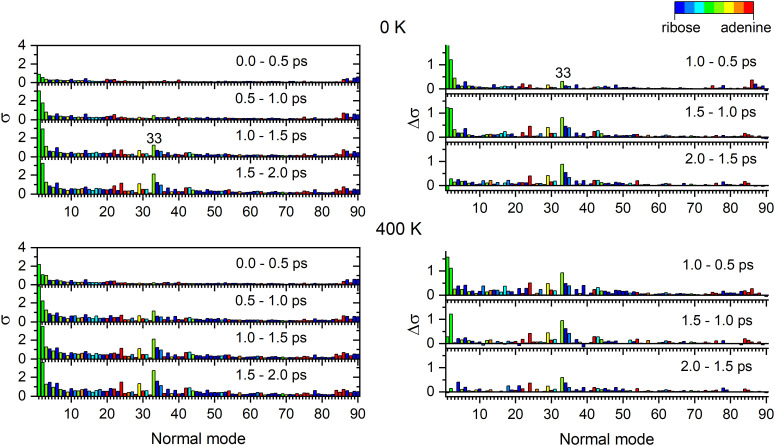
Normal mode analysis for adenosine at 0 (top) and 400 K (bottom). Each panel averages the results of all trajectories. Left: The standard deviation (*σ*) to the mean value is shown for each normal mode accumulating all data with 0.5 ps periods. Right: Standard deviation variation (Δ*σ*) between two consecutive periods. Normal modes are numbered from lower to bigger wavenumbers and colored according to the contribution of each moiety.

Fig. 7 shows the total standard deviation (left side) and the change from period to period (right side). For example, in the top left graph, “0.0–0.5 ps” means the standard deviation for time steps within the first 0.5 ps of dynamics. In the graph on the top right, “1.0–0.5 ps” means how much the standard deviation changed from the first to the second period. Thus, we can detect which modes are receiving energy during the dynamics. The graphs are colored with a partition of the normal mode between ribose and adenine, where blue represents ribose only, red represents adenine only, and green indicates the mixed contribution from both, ribose and adenine groups.

For both 0 and 400 K, the evolution is similar. Modes 1, 2, and 33 have the largest standard deviation and also the most significant standard deviation variations. Thus, they should be the modes receiving the energy from the excited-state relaxation. These three modes mix contributions from adenine and ribose. Thus, the energy transfer to ribose occurs through coupled adenine-ribose motions. Mode 1 receives energy only in the first picosecond, while mode 2 stops receiving energy after 1.5 ps. Mode 33 remains active until later times.

**Fig. 8:**
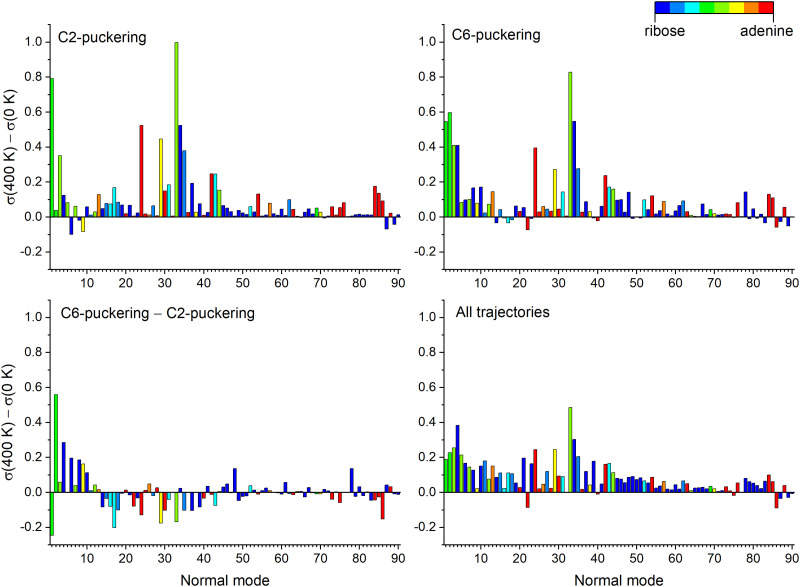
Normal mode analysis of adenosine integrated over 2 ps. Each graph shows the difference between the standard deviation of each mode at 0 and 400 K [*σ*(400)–*σ*(0)] for four cases. Top left: Only trajectories with C2-puckering S_1_/S_0_ internal conversion within 2 ps are considered. Top right: Only trajectories with C6-puckering S_1_/S_0_ internal conversion within 2 ps are considered. Bottom left: Difference between the two previous cases (C6–C2). Bottom right: all trajectories are considered.

Fig. 8 focuses on how the thermal energy affects the normal modes when adenosine is heated from 0 to 400 K. In the trajectories returning to S_0_ through C2 puckering (top left), Modes 33, 1, 24, and 34 (in decreasing importance order) receive the most energy. Among those trajectories returning through C6 puckering (top right), the most relevant normal modes are 33, 2, 1, and 34. The difference between the C6 and C2, shown in the bottom left, confirms the importance of Mode 2 for C6-puckering trajectories. In the ensemble of all trajectories (bottom right), heating most activates Modes 33 and 4.

## 5. Conclusion

We employed surface hopping to simulate the nonadiabatic dynamics of photoexcited adenine and adenosine at 0 and 400 K. The goal was to investigate the dependence of excited-state relaxation on the temperature. We observed that the excited-state lifetime of adenosine is 2.3 times longer at 0 K compared to the lifetime at 400 K. In contrast, the lifetime of adenine does not depend on the temperature. This result is in line with the experimental findings of West *et al.*^4^

The nonadiabatic dynamics of both molecules at 0 K and 400 K followed the usual ring puckering mechanisms. The main distinction was the intramolecular vibrational energy transfer from adenine to the ribose group in adenosine. For adenosine at 0 K, this energy transfer reduced the mean kinetic energy of adenine's moiety to the point that internal conversion was inhibited. On the other hand, in adenine (at both temperatures) and adenosine at 400 K, the mean kinetic energy in the adenine moiety was large enough to reach the state intersection within similar times.

Normal mode analysis revealed that three modes dominate adenosine's vibrational energy transfer. They are two coupled adenine-ribose torsions with low wavenumbers of about 40 cm^−1^ and coupled adenine-ribose bending with a wavenumber of 755 cm^−1^. Comparing dynamics at 0 and 400 K shows that two normal modes are notably relevant during the excited state relaxation, receiving much thermal energy. They are the adenine-ribose bending at 755 cm^−1^ and the alkyl hydroxy twist at 102 cm^−1^.

These results settle the discussion on whether the temperature dependence of adenosine's excited-state lifetime was caused by intramolecular energy transfer or the medium viscosity, favoring the former explanation.

## Author contributions

Ritam Mansour: investigation; formal analysis; visualization; writing – original draft; Josene M. Toldo: investigation; formal analysis; data curation; visualization; writing – original draft; writing – review & editing; Saikat Mukherjee: formal analysis; Max Pinheiro Jr: software; Mario Barbatti: conceptualization; methodology; software; visualization; supervision; writing – review & editing; funding acquisition.

## Conflicts of interest

There are no conflicts to declare.

## Supplementary Material

CP-025-D3CP03234E-s001
