# Supplementary material for: Temperature effects on the internal conversion of excited adenine and adenosine
Source: Phys Chem Chem Phys. 2023 Sep 22;25(40):27083–93. doi: 10.1039/d3cp03234e (PMC10583498; doi:10.1039/d3cp03234e)
Supplement: CP-025-D3CP03234E-s001 [file CP-025-D3CP03234E-s001.pdf]

Electronic Supplementary Material (ESI) for Physical Chemistry Chemical Physics.
This journal is © the Owner Societies 2023

Electronic Supplementary Information

Temperature effects on the internal conversion of excited adenine and adenosine

Ritam Mansour,[1] Josene M. Toldo,[1*] Saikat Mukherjee,[1] Max Pinheiro Jr,[1] Mario Barbatti[1,2*]

[1] *Aix-Marseille University, CNRS, ICR, Marseille, France*
[2] *Institut Universitaire de France, 75231, Paris, France*

Table of Contents

SI-1. EXCITED-STATE CHARACTERIZATION 2
SI-2. NUCLEAR-ENSEMBLE SPECTRA 5
SI-3. EXCITED-STATE OCCUPATION 6
SI-4. ELECTRONIC DENSITY DIFFERENCES 6
SI-5. NORMAL MODE ANALYSIS 7
SI-6. ACTIVE NORMAL MODES 8
SI-7. SUPPLEMENTARY REFERENCES 9

SI-1. Excited-state characterization

Table S1. Excitation energies, oscillator strengths (*f*), and state characterization given by natural transition orbitals and their respective contributions (in percentage) calculated for 9H-adenine and *anti*-adenosine at the S_0 minimum at ADC(2)/SV(P) level.

	Adenine			Adenosine		
**State**	Energy (eV)	*f*	State characterization	Energy (eV)	*f*	State characterization
**S_1**	5.16	0.001	nπ* (0.96)	5.13	0.000	nπ* (0.95)
**S_2**	5.34	0.060	ππ* (0.71)<br>ππ* (0.26)	5.25	0.091	ππ* (0.74)<br>ππ* (0.21)
**S_3**	5.52	0.269	ππ* (0.89)	5.45	0.283	ππ* (0.86)

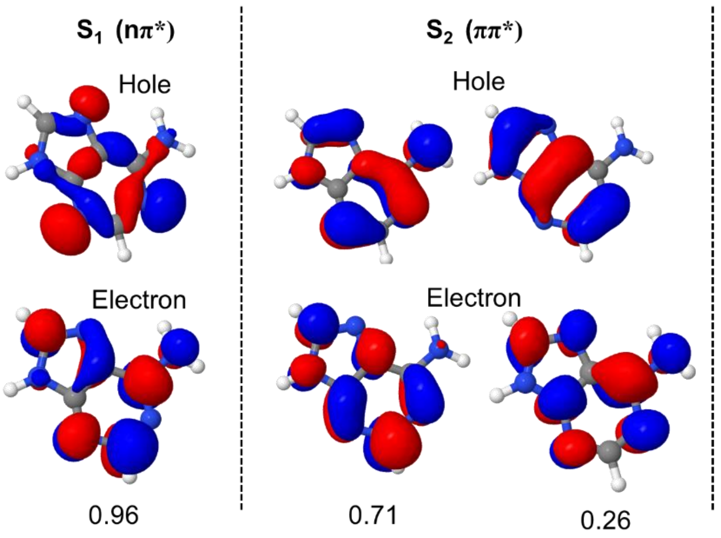

Figure S1. Natural transition orbitals (NTOs) describing the first singlet excitations on 9H-adenine at the ground state geometry. NTOs computed at ADC(2)/SV(P) level.

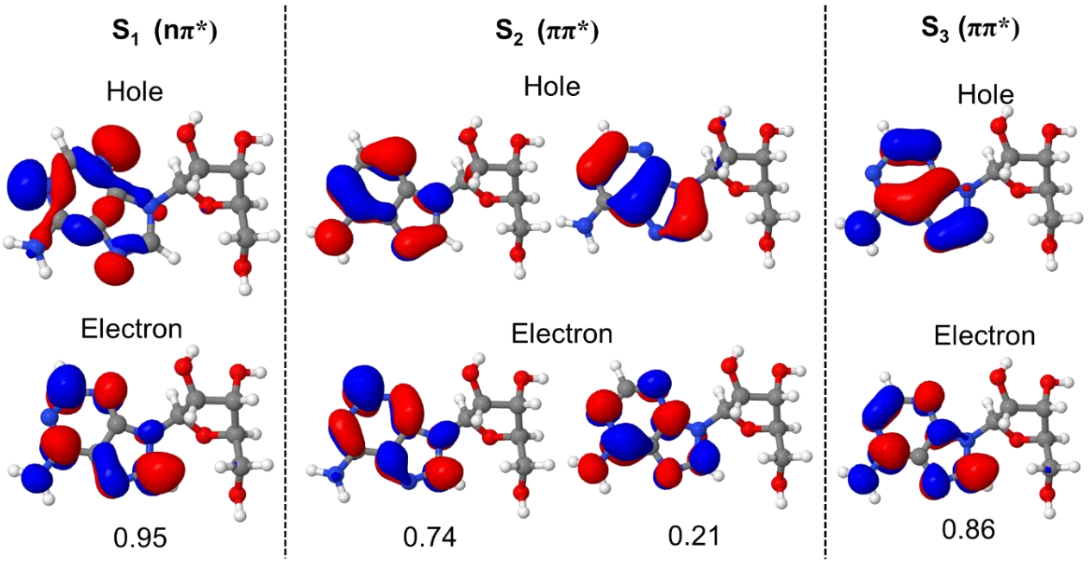

Figure S2. Natural transition orbitals (NTOs) describing the first singlet excitations on *anti*-adenosine at the ground state geometry. NTOs computed at ADC(2)/SV(P) level.

Vertical excitations and potential energy surfaces of adenine were explored at different levels of theory (see Table S2 and supplementary Ref. 1). Reaction pathways connecting ground state minimum and conical intersections for adenine at the multiconfigurational level are also explored in previous works.[2-4] It has been shown that ADC(2) can satisfactorily reproduce both vertically excited states and reaction pathways for adenine.

Table S2. Comparison of excitation energies and oscillator strengths (*f*) obtained at different levels of theory for 9H-adenine. L_b and L_a states denote, respectively, the weakly absorbing ππ* state and the bright ππ* state.

**9H-Adenine**				
**Level of theory**	**nπ***	**ππ* (L_b)**	**ππ* (L_a)**	**Reference**
RI-ADC(2)/SV(P)	5.16 (0.001)	5.34 (0.060)	5.52 (0.269)	This work.
RI-ADC(2)/aug-cc-pVDZ [a]	5.00 (0.03)	5.07 (0.10)	5.06 (0.17)	Aquino *et al.*[5]
CC2/aug-cc-pVTZ [b]	5.12 (0.01)	5.25 (0.02)	5.25 (0.30)	Fleig *et al.*[6]
MS-CASPT2(12,10)/ANO-L [c]	4.85	5.09	5.44 (0.18)	Conti *et al.*[7]
MS-CASPT2/aug-cc-pVTZ [d]	5.04 (0.02)	5.02 (0.02)	5.12 (0.40)	Silva-Junior et al.[8]
MS-CASPT2(16,12)/6-31G(d) [e]	5.29	5.43	5.56	Barbatti *et al.*[9]
MS-CASPT2(16,12)/6-31G(d) [f]	4.96	5.03	5.17	Barbatti *et al.*[9]
MS-CASPT2(16,13)/6-31G(d) [g]	4.96	5.16	5.35	Serrano-Andrés *et al*[10]
MS-CASPT2/6-31+G(d) [h]	5.31 (0.010)	5.42 (0.039)	5.61 (0.152)	Barbatti *et al.*[4]
MS6-CASPT2(14,10)/VDZ [i]	4.86	5.13	5.28	Marchetti *et al.*[3]
Experimental (gas phase) [j]			5.16 ± 0.07 (0.24)	Barbatti *et al.*[4]
Experimental (H_2O) [l]			4.76	Voet *et al.*[11]

[a]GS geometry optimized at MP2/TZVP level in C1 point-group symmetry. [b] GS geometry optimized at CC2/aug-cc-pVTZ level in C1 point group symmetry. [c]CASPT2//CASSCF(12,10)/ANO-L state averaged (SA) on six roots. GS geometry optimized at CASSCF(8,8)/6-31G* level of theory. [d] GS geometry optimized at MP2-6-31G* in Cs point group symmetry, SA-8 for ππ* states, and SA-3 for nπ* states. Active space (0 13)12 for ππ* and (3 13)18 for nπ* (active space orbitals of A′ and A″ symmetry given in parentheses followed by the number of electrons). [e]CASPT2/SA5-CASSCF(16,12)/6-31G* method used for single-point calculations on geometries optimized at the MR-CIS(6,5)/SA-3-CAS(12,10)/6-31G* level; IPEA shift of 0.25. [f] CASPT2/SA5-CASSCF(16,12)/6-31G* method used for single-point calculations on geometries optimized at the MR-CIS(6,5)/SA-3-CAS(12,10)/6-31G* level; IPEA 0.00. [g]CASPT2//CASSCF(16,13)/6-31G* method. [h]CAS(18,13); Level shift 0.2 a.u; IPEA 0.25 a.u. [i]Geometry optimization with CASSCF(10,8)/VDZ and energies calculated with MS6-CASPT(14,10)/VDZ. [j] Estimated from gas phase band maximum data. [l] Data from UV spectra in water, pH 7.0.

Adenosine vertical excitation energies resemble the ones from adenine, as shown in our ADC(2) calculations and also experimentally[11] (see Tables S2 and S3). Similarly to adenine, the S_1 state of adenosine has a nπ* character at the Franck-Condon region. For the syn-isomer, CASPT2 calculations also identify a ππ*/S_0 MECI with a puckered geometry lying lower than the S_1 state at the Franck-Condon region. EDPT reaction pathway from the bright ππ* (L_a) state to this intersection is barrierless, but from the S_1 (nπ*) to the ππ*/S_0 MECI, a barrier of 0.3 eV is observed.[3] Reaction pathways computed at both ADC(2) and CASPT(2) levels for adenosine (Figure 15 and Figure S34 of Ref. 3) show that ADC(2) can reproduce the main features.

Table S3. Comparison of excitation energies and oscillator strengths (*f*) obtained at different levels of theory for adenosine. L_b and L_a states denote, respectively, the weakly absorbing ππ* state and the bright ππ* state.

**Adenosine**				
**Level of theory**	**nπ***	**ππ* (L_b)**	**ππ* (L_a)**	**Reference**
RI-ADC(2)/SV(P)	5.13 (0.001)	5.25 (0.060)	5.45 (0.269)	This work
ADC(2)/cc-pVDZ	5.20	5.18	5.30	Tuna et al.[12]
MS6-CASPT2(10,10)/VDZ[a]	5.0	5.55	5.65	Marchetti *et al.*[3]
Experimental (H_2O)[j]			4.78	Voet *et al.*[11]

[a] Optimizations with MP2/6-31G(d) and vertical energies calculated with MS6-CASPT2(10,10)/VDZ. [j] Data from UV spectra in water, pH 7.9.

SI-2. Nuclear-ensemble spectra

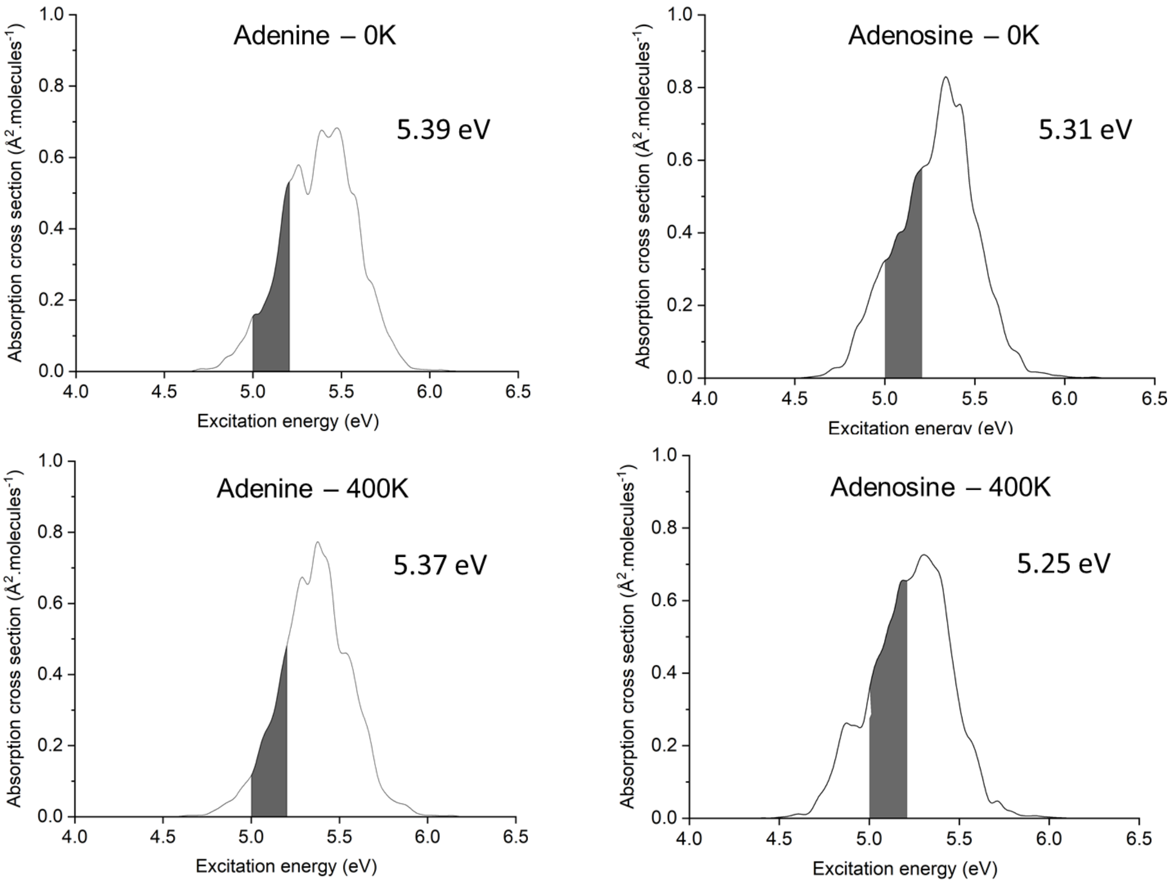

Figure S3. Computed absorption spectra based on 500 points nuclear ensemble for adenine and adenosine at 0 and 400 K calculated at ADC(2)/SV(P) level. The grey area represents the excitation window (5.1 ± 0.1 eV in all cases).

SI-3. Excited-state occupation

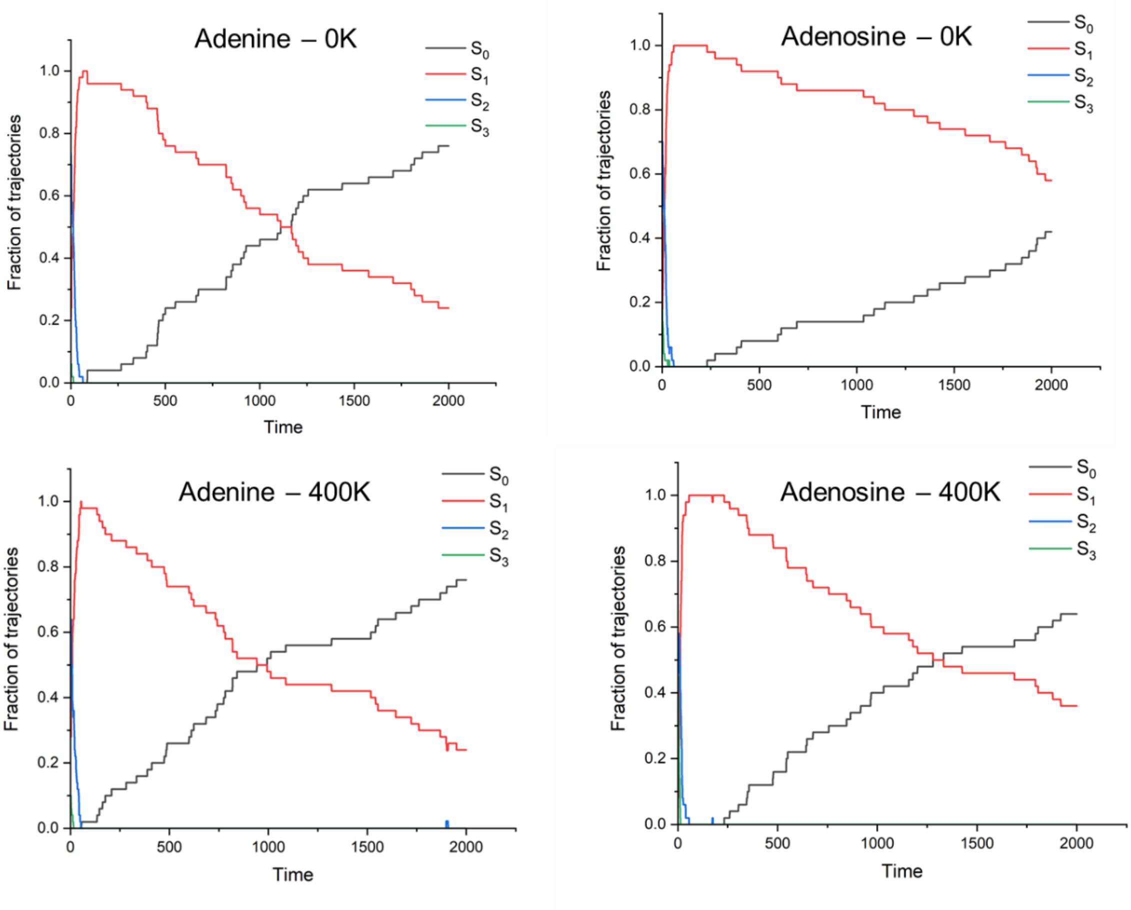

Figure S4. Adiabatic state occupation evolution for adenine and adenosine at 0 and 400 K calculated at ADC(2)/SV(P) level.

SI-4. Electronic density differences

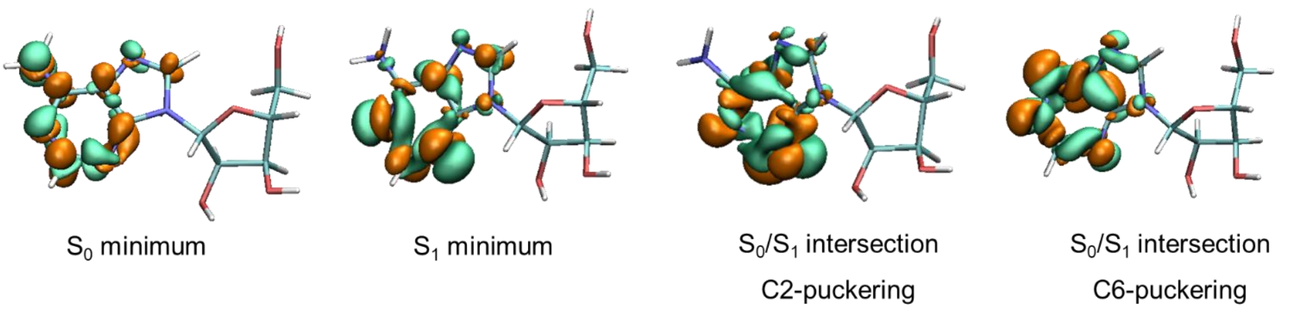

Figure S5. Electronic density difference between S_1 and S_0 states at the S_0 minimum, S_1 minimum, C2-puckered and C6-puckered intersections. Green and orange regions represent a decrease and an increase in electron density, respectively.

SI-5. Normal mode analysis

Normal modes are linear combinations of cartesian coordinates, typically obtained as the eigenvectors of the mass-weighted Hessian matrix of the energy. They are handy in identifying relevant nuclear vibrational motions of polyatomic molecules. The normal mode analysis is a way to project the molecular motion of a trajectory in terms of the corresponding normal modes displacement of the molecule.[13, 14]

Let $\boldsymbol{Q} = (\boldsymbol{q_1}|\boldsymbol{q_2}|\cdots|\boldsymbol{q_{3N-6}})$ be the matrix containing the $3N-6$ normal modes at a reference geometry $\boldsymbol{R}_0$ in Cartesian coordinates, where N is the number of atoms in a molecule. $\boldsymbol{R}(i,t)$ is the Cartesian coordinates of the ith trajectory at time t. The displacement vector in normal mode coordinates (i.e., normal mode amplitudes) with respect to the reference geometry can be written as

$$\boldsymbol{\Omega}(i,t) = \boldsymbol{Q}^{-1}[\boldsymbol{R}(i,t) - \boldsymbol{R}_0]$$

Taking an average over all trajectories, the average displacement $\bar{\boldsymbol{\Omega}}(t)$ for each normal mode can be obtained to monitor the average motion (nuclear vibrations) of the molecule as a function of time. Moreover, the standard deviation of this average displacement over time boils down to a single number per normal mode to represent its coherent activity:

$$\widehat{\boldsymbol{\Omega}}_{coh}^2 = \frac{1}{k_{fin} - k_{ini}} \sum_{k=k_{ini}}^{k_{fin}} \bar{\boldsymbol{\Omega}}(k\Delta t)^2 - \left(\frac{1}{k_{fin} - k_{ini}} \sum_{k=k_{ini}}^{k_{fin}} \bar{\boldsymbol{\Omega}}(k\Delta t) \right)^2$$

In this equation, $\widehat{\boldsymbol{\Omega}}_{coh}^2$ shows the coherent activity of a normal mode during the dynamics. k_{ini} and k_{fin} are the first and last time steps considered, and Δt is the constant time interval of the dynamics.

Alternatively, one may compute the total standard deviation over time steps and trajectories without prior averaging over the trajectories. Hence, it will quantify the total motion observed along the normal modes.

$$\widehat{\boldsymbol{\Omega}}_{i,total}^2 = \frac{1}{k_{fin} - k_{ini}} \sum_{k=k_{ini}}^{k_{fin}} \boldsymbol{\Omega}(i,k\Delta t)^2 - \left(\frac{1}{k_{fin} - k_{ini}} \sum_{k=k_{ini}}^{k_{fin}} \boldsymbol{\Omega}(i,k\Delta t) \right)^2$$

Because we aim to describe the total motion, our results discussed in the paper correspond to this second quantity.

SI-6. Active normal modes

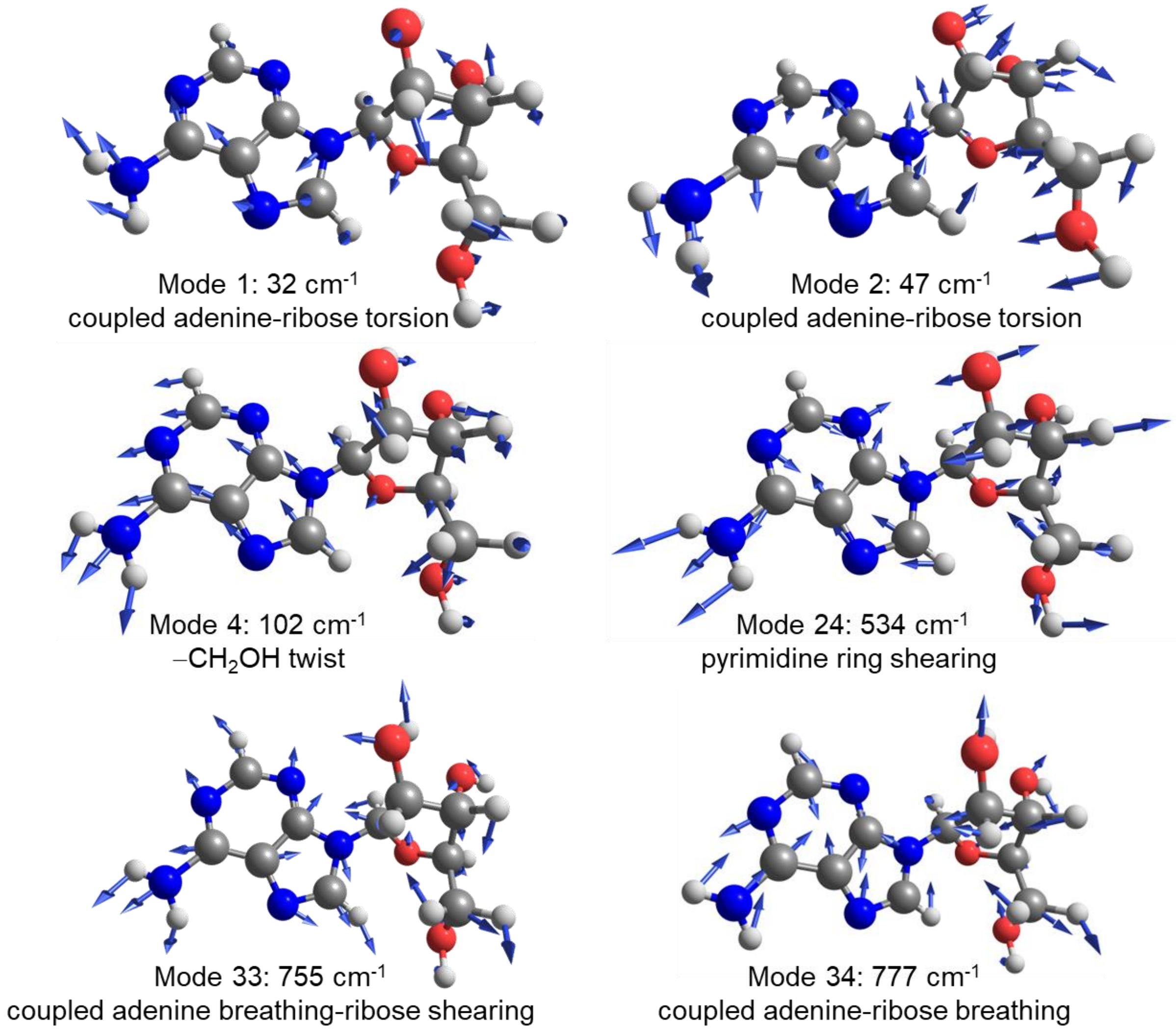

Figure S6. Main normal modes contributing to vibrational energy transfer in adenosine.

SI-7. Supplementary references

1. R. Improta, F. Santoro and L. Blancafort, *Chem. Rev.*, 2016, **116**, 3540-3593. https://doi.org/10.1021/acs.chemrev.5b00444
2. F. Plasser, R. Crespo-Otero, M. Pederzoli, J. Pittner, H. Lischka and M. Barbatti, *J. Chem. Theory Comput.*, 2014, **10**, 1395-1405. https://doi.org/10.1021/ct4011079
3. B. Marchetti, T. N. V. Karsili, M. N. R. Ashfold and W. Domcke, *Phys. Chem. Chem. Phys.*, 2016, **18**, 20007-20027. https://doi.org/10.1039/C6CP00165C
4. M. Barbatti and S. Ullrich, *Phys. Chem. Chem. Phys.*, 2011, **13**, 15492-15500. https://doi.org/10.1039/C1CP21350D
5. A. J. A. Aquino, D. Nachtigallova, P. Hobza, D. G. Truhlar, C. Hättig and H. Lischka, *J. Comput. Chem.*, 2011, **32**, 1217-1227. https://doi.org/https://doi.org/10.1002/jcc.21702
6. T. Fleig, S. Knecht and C. Hättig, *J. Phys. Chem. A*, 2007, **111**, 5482-5491. https://doi.org/10.1021/jp0669409
7. I. Conti, P. Altoè, M. Stenta, M. Garavelli and G. Orlandi, *Phys. Chem. Chem. Phys.*, 2010, **12**, 5016-5023. https://doi.org/10.1039/B926608A
8. M. R. Silva-Junior, S. P. A. Schreiber M Fau - Sauer, W. Sauer Sp Fau - Thiel and W. Thiel.
9. M. Barbatti and H. Lischka, *J. Am. Chem. Soc.*, 2008, **130**, 6831-6839. https://doi.org/10.1021/ja800589p
10. L. Serrano-Andrés, M. Merchán and A. C. Borin, *Proc. Natl. Acad. Sci. USA*, 2006, **103**, 8691-8696.
11. D. Voet, W. B. Gratzer, R. A. Cox and P. Doty, *Biopolymers*, 1963, **1**, 193-208. https://doi.org/https://doi.org/10.1002/bip.360010302
12. D. Tuna, A. L. Sobolewski and W. Domcke, *J. Phys. Chem. A*, 2014, **118**, 122-127. https://doi.org/10.1021/jp410121h
13. L. Kurtz, A. Hofmann and R. de Vivie-Riedle, *J. Chem. Phys.*, 2001, **114**, 6151-6159. https://doi.org/10.1063/1.1355658
14. F. Plasser, M. Barbatti, A. J. A. Aquino and H. Lischka, *J. Phys. Chem. A*, 2009, **113**, 8490-8499. https://doi.org/10.1021/Jp9032172
